# Supplementary material for: Antibacterial Activity of Silver-Modified CuO Nanoparticle-Coated Masks
Source: Bioengineering (Basel). 2024 Dec 5;11(12):1234. doi: 10.3390/bioengineering11121234 (PMC11672906; doi:10.3390/bioengineering11121234)
Supplement: Supplementary file 1 [file bioengineering-11-01234-s001.zip › bioengineering-3278366-supplementary.pdf]

Electronic Supplementary Information

Antibacterial Activity of Silver-Modified CuO Nanoparticle-Coated Masks

Tanuja Udawant [1], Prajkta Thorat [1], Payal Thapa [2], Manali Patel [2], Saroj Shekhawat [2], Roshni Patel [2], Ankit Sudhir [2], Om Hudka [3], Indra Neel Pulidindi [4,*] and Archana Deokar [1,3,*]

1 Modern College of Arts, Science, and Commerce, Pune 411005, India; tanujaudawant159@gmail.com (T.U.); prajaktathorat03@gmail.com (P.T.)

2 Department of Life Sciences, School of Science, Gujarat State Fertilizers and Chemicals University, Vadodara 391750, India; 23msc02019@gsfcuniversity.ac.in (P.T.); 23msc02005@gsfcuniversity.ac.in (M.P.); saroj.shekhawat@gsfcuniversity.ac.in (S.S.); roshni.patel@gsfcuniversity.ac.in (R.P.); ankit.sudhir@gsfcuniversity.ac.in (A.S.)

3 Department of Chemical Sciences, School of Science, Gujarat State Fertilizers and Chemicals University, Vadodara 391750, India; 22msc01014@gsfcuniversity.ac.in

4 Jesus' Scientific Consultancy for Industrial and Academic Research (JSCIAR), Tharamani 600113, India

* Correspondence: indraneelp@jesusconsultancy.com (I.N.P.); archana.deokar@gsfcuniversity.ac.in (A.D.); Mobile.: +91-9597925504 (I.N.P.)

Table S1. Zone of inhibition (ZoI) by Ag-CuO NPs against gram-positive *S. aureus* and gram-negative *E. coli.*

**Bacteria (10^6 cfu/mL)**	**ZoI as a function by Ag -CuO NPs as a function of bacterial strain**	
	**Control**	**5000 ppm**
S. aureus	**0**	**3.88 ± 0.23**
E. coli	**0**	**3.24± 0.02**

The ZoI method was employed to evaluate the efficacy of synthesized Ag-CuO NPs against gram-positive (*S. aureus*) and gram-negative (*E. coli*) bacterial strains. Table S1 represents the ZoI observed against differnet bacterial strains with a Ag-CuO NPs concentraiton of 5000 ppm. It is observed that Ag-CuO NPs showed outstanding antibacterial activity. An optimal concentration (5000 ppm) of NPs demonstrated an antibacterial activity against both *S. aureus* (***3.88 ± 0.23 mm***) and *E. coli* (***3.24 ± 0.02 mm***) probably due to the production of ROS. This is in accordance with our previous study [12]. It has been proved in our earlier study that zinc doped CuO NPs produces higher ROS compared to native metal oxides (CuO/ZnO NPs) making them effective antimicrobial material for killing microbes. Also, the role of ROS production was extensively investigated. In particular the generation of OH· radicals and superoxide anion ($^{\bullet}O_2^-$), as a primary cytotoxic mechanism exerted by metal oxide NPs against bacteria was examined. The oxidative stress, stemming from ROS, induces significant damage to the cell membrane, ultimately resulting in cell death. Moreover, it has been proven that silver

NPs possesses superior antimicrobial activity owing to their ability to release silver ions (Ag^+) in the presence of moisture, such as body fluids or water. Owing to the synergitic effect of both Ag and CuO NPs, an enhanced antibacterial activity is observed with the specially desgined masks coated with Ag-CuO NPs for eradicating bacteria, namely, *S . aureus* and *E. coli*.

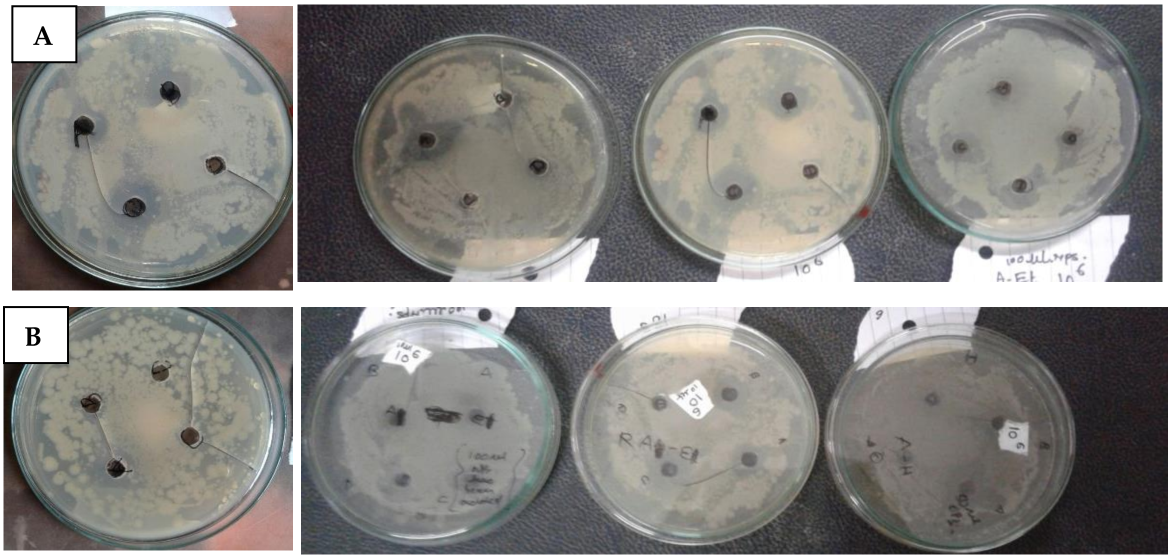

Figure S1. Zone of inhibition studies of silver modified copper oxide nanoparticles against (A) *E.coli* and (B) *S. aureus*
